# The Rise of Total-Body PET/CT: Advancing Molecular Imaging Toward Early Cancer Detection and Potential Future Application in Prevention Healthcare

**DOI:** 10.3390/jcm15010311

**Published:** 2025-12-31

**Authors:** Pierpaolo Alongi, Simone Morea, Roberto Cannella, Rosa Alba Pugliesi, Carlo Messina, Daniele Di Biagio

**Affiliations:** 1 Department of Biomedicine, Neurosciences and Advanced Diagnostics, University of Palermo, Piazza Marina 61, 90139 Palermo, Italy; roberto.cannella@unipa.it (R.C.); rosaalba.pugliesi@unipa.it (R.A.P.); 2 Nuclear Medicine Centre, San Pietro Fatebenefratelli Hospital—Express Diagnostics, Via Cassia 600, 00189 Rome, Italy; s.morea@expressdiagnostic.it (S.M.); d.dibiagio@expressdiagnostic.it (D.D.B.); 3 Oncology Unit, UOC Oncologia ARNAS Civico Benfratelli di Cristina, Piazza Nicola Leotta 4, 90127 Palermo, Italy; carlo.messina@arnascivico.it

**Keywords:** positron emission tomography, LAFOV, ultralow-dose, surveillance, total body, cancer

## Abstract

Positron Emission Tomography (PET) is undergoing a profound transformation. Driven by the convergence of highly sensitive long-axial field-of-view (LAFOV) total-body PET systems and an expanding portfolio of targeted radiopharmaceuticals, PET is progressively evolving beyond its traditional role in oncologic diagnosis and staging. Ultra-sensitive scanners enable whole-body imaging with markedly reduced radiotracer doses, rapid acquisition times, and true dynamic multiparametric imaging across all organs simultaneously. In parallel, molecularly targeted radioligands support tumour phenotyping, theranostic applications, and personalized dosimetry. Together, these advances position PET as a systemic imaging platform capable of interrogating whole-body tumour biology, guiding precision therapies, and potentially enabling early detection or surveillance strategies in selected high-risk populations. This narrative review summarizes the technological foundations of total-body PET, reviews current clinical and translational applications, discusses opportunities and limitations for early detection and surveillance, and outlines a research and implementation roadmap to responsibly translate this paradigm into clinical oncology.

## 1. Introduction

For decades, positron emission tomography (PET) has distinguished itself from conventional radiological modalities by visualizing metabolic and molecular activity, rather than relying solely on anatomical changes [1]. Radiotracers such as ^18^F-Fluorodeoxiglucose (^18^F-FDG) enabled clinicians to identify hypermetabolic lesions, guide staging, assess therapeutic response, and plan radiation or systemic therapies [2]. Over time, the introduction of receptor-targeted or phenotype-specific radioligands (e.g., for somatostatin receptors in neuroendocrine tumors, or prostatic specific membrane antigene—PSMA in prostate cancer) further extended PET’s diagnostic and prognostic utility [3]. However, these applications were bounded by fundamental limitations: the restricted axial field-of-view (FOV) of PET scanners—typically 15–32 cm—constrained the extent of the body that could be imaged in a single pass; sensitivity was relatively limited if compared with new technology; acquisitions often required doses of radiotracer that limit the diffusion of the application [4]; dynamic or repeated whole-body imaging was impractical.

The recent advent of total-body long-axial-field-of-view (LAFOV) PET scanners—capable of covering nearly the entire adult human body (e.g., an axial FOV of 194 cm) in a single acquisition—represents a qualitative advancement [5,6]. This structural leap, combined with modern radioligands and numerical methods, challenges the classical boundaries of PET and invites its re-conceptualization. What was once niche and reactive may become systemic and anticipatory. We argue that we stand at a pivotal moment: PET may evolve into a foundational platform for systemic oncology—potentially supporting theranostics, personalized therapy, pharmacokinetics, and even early detection or, hopefully in a near future, for surveillance in high-risk populations.

Clinical studies have demonstrated that ultralow-dose protocols on LAFOV-PET/CT preserve quantitative robustness and lesion detectability while minimizing radiation exposure, which is particularly relevant for radiation-sensitive populations and repeated imaging scenarios. Beyond dose and image quality benefits, LAFOV PET/CT optimizes patient workflow by reducing scanning time and decreasing patient-reported anxiety and radiation concerns, without increasing physical discomfort, thereby supporting both clinical efficiency and patient-centered care.

This narrative review aims to contextualize these advances, critically discuss their clinical implications, and explore the emerging hypothesis that total-body PET may ultimately support earlier cancer detection or surveillance in carefully selected populations.

## 2. Materials and Methods

This article was conducted as a narrative review of the literature with a vision on perspective on future potential development of new era of PET technology. A non-systematic search of PubMed (National Library of Medicine, Bethesda, MD), Scopus(Elsevier), and major nuclear medicine journals was performed to identify relevant publications up to November 2025 addressing total-body PET/CT technology, LAFOV systems, radiopharmaceutical development, theranostics, ultralow-dose imaging, and potential applications in early cancer detection or surveillance. Search terms included combinations of “total-body PET”, “LAFOV PET”, “uultralowdose PET”, “theranostics”, “molecular imaging”, and “cancer surveillance”.

Original research articles, technical performance studies, clinical trials, meta-analyses, and authoritative reviews were preferentially considered. Reference lists of key articles were manually screened to identify additional relevant studies. Given the narrative nature of the review, study selection was based on relevance, methodological quality, and contribution to the conceptual framework rather than formal quantitative synthesis.

## 3. The Technological Leap: What Total-Body PET Enables

The first human images acquired with the 194 cm axial-FOV PET/CT scanner provided evidence of possibilities long hypothesized in simulation studies [5]. The new uEXPLORER (United Imaging Healthcare, Shanghai, China) total-body PET/CT scanner is characterized by an ultra-extended axial field of view of 194 cm and a transaxial field of view of 68.6 cm, achieved through an eight-ring axial architecture with minimal inter-ring gaps, and coupled with an integrated 80-row CT system. The use of high-density LYSO detector crystals, silicon photomultiplier readout, and advanced time-of-flight capability (~430 ps) enables high sensitivity and spatial resolution (~2.9 mm at 1 cm from the center of the FOV, NEMA NU-2 2018), supporting high-quality whole-body imaging within a single acquisition [5]. In the initial four examinations in the study conducted by Badawi and co-workers, whole-body ^18^F-FDG scans of diagnostic quality were acquired using as little as 25 MBq of injected activity, or in acquisition times of few minutes—orders of magnitude below conventional whole-body PET protocols that require several hundred MBq and multiple bed positions. The authors demonstrated also a potential reduction in scan times obtaining images of diagnostic quality at 37.5 s and are arguably diagnostic even at 18.75 s compared to an acquisition of 20 min. Moreover, the same device allowed for true total-body dynamic imaging with frame durations as short as one second, enabling simultaneous pharmacokinetic assessment across all major organs [5].

A detailed physical characterization of the LAFOV-PET scanner confirmed these advantages: the system demonstrated a sensitivity of approximately 174 kcps/MBq, a peak noise-equivalent count rate (NECR) of ~2 Mcps (for total-body imaging), and spatial resolution ≤ 3.0 mm FWHM near the centre of the FOV—parameters that support uniform high-quality imaging across the entire body, even under low-dose or rapid-scan conditions [7,8]. These metrics confirm that LAFOV PET systems are not merely scaled-up versions of conventional PET, but represent a fundamentally improved detection geometry with enhanced count efficiency, better signal-to-noise ratios, and increased dynamic range.

Similarly, Honoré d’Este et al., using another 106 cm LAFOV-PET system, demonstrated that using an administered activity of 3 MBq/kg of [18F]FDG, diagnostically acceptable image noise, lesion detectability, and lesion classification can be achieved with markedly reduced acquisition times. Quantitative and qualitative image quality reached current standards of care after 90 s, with further significant improvements up to 180–300 s, supporting whole-body imaging protocols of less than 5 min on LAFOV systems [9].

**Figure 1:**
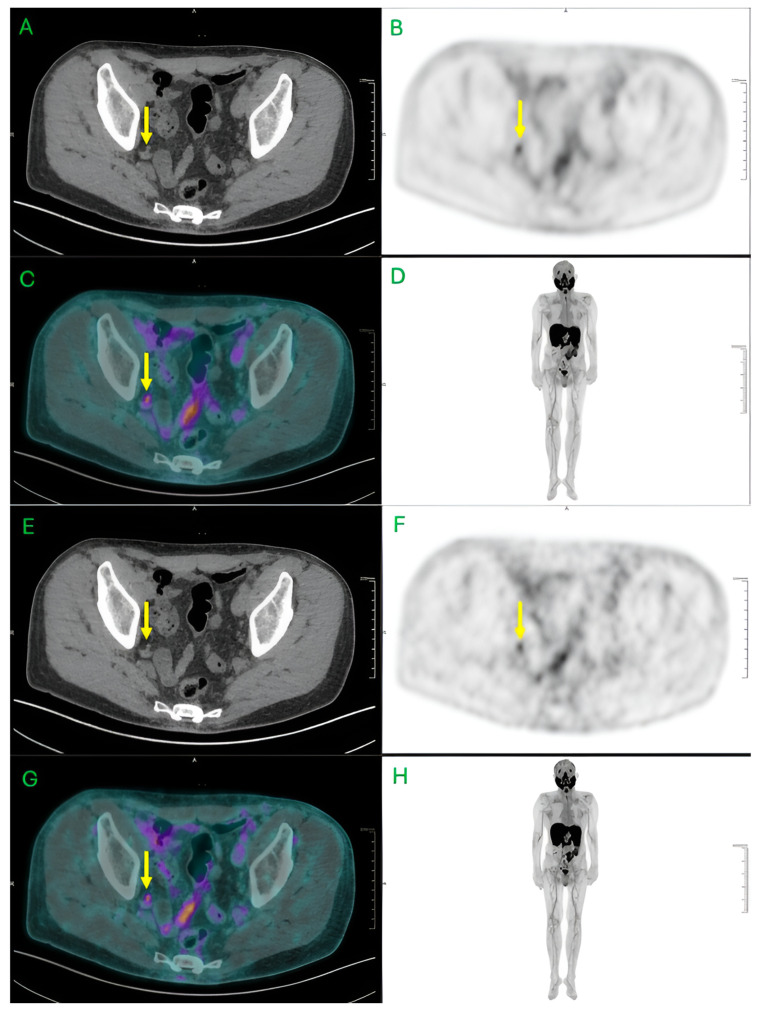
Preservation of high sensitivity using fast acquisition protocol on uEXPLORER Total Body PET/CT. LAFOV Total Body PET/CT (194 cm-uEXPLORER, United Imaging, Shangai, China) with 18F-Piflufolastat (190 MBq) in the restaging process of prostate cancer patient with biochemical relapse after prostatectomy (PSA 0.63 ng/mL). (**A**–**D**) represent axial PET/CT images and Maximum Intensity Projection (MIP) from standard acquisition protocol (1 bed of 8 min); Yellow Arrow on a 4 mm right internal iliac nodal disease with mild activity (SUVmax 2.23; Tumor Background Ratio—TBR = 7.43). Images (**E**–**H**) show similar qualitative PET/CT images from a post-processing reconstruction just for 1 min acquisition (SUVmax 2.2; TBR = 8.14). Images provided by Nuclear Medicine Centre, San Pietro Fatebenefratelli Hospital, Rome, Italy.

As a result, LAFOV PET fundamentally redefines the trade-offs between injected activity, image quality, scan time, and coverage [4] (Figure 1). Clinicians and researchers can now choose among different protocols: low-dose whole-body imaging (ideal for surveillance or repeated studies), rapid acquisition (for throughput and patient comfort), delayed or late imaging (for tracer kinetics), or high-resolution dynamic studies (for pharmacokinetics or dosimetry) [9,10]. This flexibility positions total-body PET not only as a diagnostic tool but as a platform for systemic biology, dosimetry, and repeated longitudinal imaging.

## 4. Radiopharmaceutical Innovation and Theranostics: Matching Molecular Targets with High-Sensitivity Imaging and Dynamic Acquisition

While hardware has evolved dramatically, radiopharmaceutical development has kept pace. The expansion of molecularly targeted tracers permits PET to probe tumour biology at a receptor, transporter, metabolic, or microenvironmental level—far beyond glucose metabolism [3]. Examples include ligands for prostate-specific membrane antigen (PSMA), somatostatin receptors (SSTR) in neuroendocrine tumours (NETs), amino-acid tracers (e.g., for brain tumours), fibroblast activation protein (FAP) inhibitors, and emerging immuno-PET agents targeting immune checkpoints or other tumour microenvironment markers [11,12,13]. Meta-analyses and reviews have highlighted that beyond FDG, such tracers substantially improve tumour detection, characterization, and specificity across multiple cancer types [2,11,12,13].

Of special relevance is the theranostic paradigm: imaging and therapy sharing the same molecular target. SSTR-targeted PET coupled with peptide receptor radionuclide therapy (PRRT) using ^177^Lu-DOTATATE is now standard of care in many NETs, based on randomized evidence that showed significant progression-free survival benefit and acceptable long-term safety [14,15]. Similarly, PSMA-directed radioligand therapy for prostate cancer builds upon PSMA PET/CT identification of target expression [16].

Total-body PET enhances this paradigm by offering high-sensitivity, low-dose, whole-body quantification of receptor expression or tracer uptake, improving patient selection, enabling personalized dosimetry, and facilitating response monitoring [17]. In addition, newer radiotracers developed for dosimetry (e.g., ^90^Y, ^177^Lu), immuno-PET or microenvironmental imaging increasingly might benefit from the high sensitivity and dynamic range of LAFOV systems [6]. As suggested by Slart and co-workers, in patients undergoing serial PET/CT examinations—particularly in oncology for treatment response assessment—the enhanced sensitivity of LAFOV PET/CT systems enables substantial reductions in administered activity, thereby supporting more frequent imaging for early identification of non-responders and timely adaptation of therapeutic strategies [10].

In about dynamic imaging, while conventional PET scanners are restricted by a limited axial field of view of 15–30 cm, requiring multiple bed positions that preclude simultaneous dynamic tracking across different body regions, LAFOV systems enable a comprehensive spatiotemporal monitoring of radiotracer distribution [18]. Dynamic PET acquisition with LAFOV systems is particularly transformative because it allows for advanced tracer kinetic modelling and the generation of whole-body parametric images [18,19]. Dynamic imaging can also quantify microkinetic parameters such as the net influx rate (Ki) and the volume of distribution (Vd). These parameters provide deeper biological insights into tracer transport and binding, offering potentially more accurate assessments of tumor response to treatment [18,19]. Furthermore, the high sensitivity of LAFOV scanners—up to 10 times higher than conventional systems—permits the extraction of an image-derived input function (IDIF) from large vessels like the aorta, facilitating non-invasive kinetic quantification for the entire body [18,19].

However, moving from single-organ to total-body parametric imaging introduces significant technical challenges that require sophisticated voxelwise modeling strategies. Research highlights that a single kinetic model applied to all image voxels is often insufficient due to the immense physiologic heterogeneity across different tissues and organs. To address this, studies suggest using voxelwise model selection strategies based on criteria like the Akaike information criterion (AIC) to choose the best-fitting model [18,19,20].

Another critical factor in total-body dynamic imaging is the non-negligible time delay between the blood input source and distant tissue regions. In conventional small-field-of-view scanners, this delay was often ignored, but in LAFOV imaging, the long distance between the heart or aorta and distant metastatic lesions can significantly impact kinetic quantification. Implementing voxelwise time delay correction (TDC) has been shown to be essential, particularly for lesions with high blood volume, as it helps eliminate vascular-region artifacts and improves the overall accuracy of multiparametric images [18,19,20,21]. By combining these advanced modeling techniques with the superior sensitivity of LAFOV scanners, clinicians can achieve high-quality diagnostic imaging while simultaneously reducing the administered radiotracer dose or acquisition time [19,20,21].

Furthermore, in the research setting, dose reduction seems particularly relevant for immuno-PET applications employing radiolabeled monoclonal antibodies with long-lived radionuclides (e.g., ^89^Zr, ^90^Y), where radiation burden is a limiting factor, while the increased system sensitivity also improves image quality for radionuclides characterized by low positron abundance. Moreover, lower injected activities facilitate multiparametric imaging approaches, including the sequential or combined use of multiple tracers within the same patient (molecular fingerprinting) [10].

Beyond dose-related advantages, the high sensitivity profile of LAFOV PET systems allows imaging at delayed time points, extending acquisitions over several additional physical half-lives of the radiotracer, which is particularly relevant for tracers with slow pharmacokinetics such as antibodies [10]. The extended axial field of view enables comprehensive whole-body assessment of the biodistribution and kinetics of novel radiotracers within a single acquisition, providing valuable dynamic information across multiple organs that may improve prediction of therapeutic efficacy and support radiopharmaceutical development [10].

Thus, the convergence of advanced radiopharmacy and ultra-sensitive imaging creates a powerful platform, turning PET into an integrated tool for diagnosis, biological phenotyping, therapy guidance, dosimetry, and longitudinal monitoring.

## 5. Toward Early Detection: Is PET Screening or Surveillance a Realistic Prospect?

The combination of total-body PET sensitivity and molecular specificity invites a provocative question: might PET transition from diagnosis and monitoring of known disease to a tool for early detection or surveillance in high-risk populations?

The technical feasibility is now established. Low-dose whole-body PET scans (e.g., using 25 MBq of ^18^F-FDG) with diagnostic quality have been demonstrated in healthy volunteers, with acceptable image quality and uniform whole-body coverage [5,22,23].

In a prospective ultralow-dose study conducted by Smith and co-workers on a LAFOV PET/CT system, oncology patients underwent paired whole-body [18F]FDG PET/CT examinations using standard (3.0 MBq/kg) and ultralow (0.3 MBq/kg) administered activities, with additional retrospective list-mode resampling to simulate activities down to 0.03 MBq/kg. Semiquantitative metrics (SUVmean, SUVpeak, SUVmax), quantitative repeatability, and lesion detectability were systematically assessed across different reconstruction protocols, demonstrating preserved stability of SUVmean and SUVpeak at markedly reduced activities, while increased variability of SUVmax and compromised lesion delineation emerged at the lowest simulated dose levels [23].

A ultralow-dose protocol on LAFOV-PET/CT in another study conducted by Smith et al., in two pregnant patients in the second and third trimester receiving an intravenous bolus injection of 0.3 MBq/kg radioactive 18F-FDG, demonstrated the possibility to obtain a diagnostic examination with a very low foetal exposure (radiation dose from the PET scan ranged between 0.11 and 0.44 mGy) [22].

**Figure 2:**
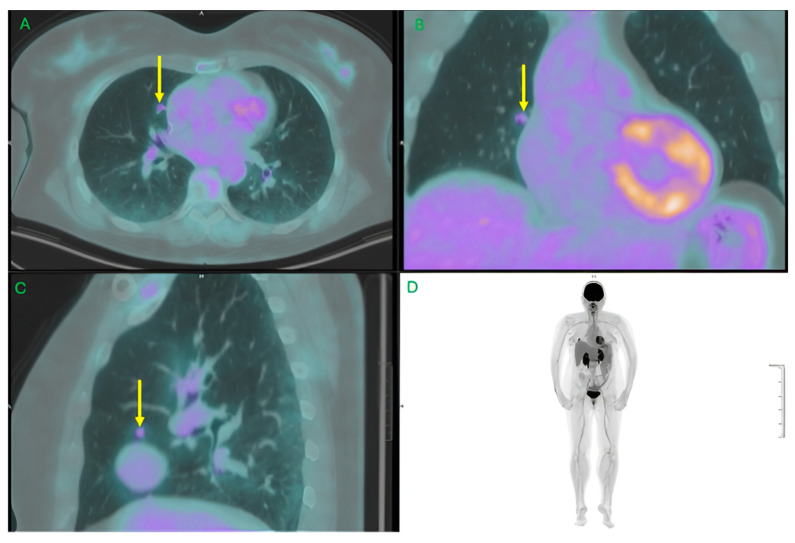
Ultralow-dose Total Body PET/CT. LAFOV-PET/CT (uEXPLORER, United Imaging) with ultralow-dose administration of 18F-FDG (69 MBq) in the staging of lung cancer, revealing rising of activity in a small lung satellite nodule (Yellow arrow; 6 mm; SUVmax 2.9; TBR = 7.25). (**A**–**C**): Axial, Coronal and Sagittal Hybrid PET/CT. (**D**): Maximum Intensity Projection (MIP). Axial images. Images provided by Nuclear Medicine Centre, San Pietro Fatebenefratelli Hospital, Rome, Italy.

This lowers a critical historical barrier—radiation dose—that limited the use of PET in repeated or screening contexts. Figure 2 shows a case presentation using LAFOV-PET/CT with ultralow-dose administration of 18F-FDG, revealing rising of activity in small lung metastatic lung nodule.

Biological rationale supports the concept: molecular changes—altered metabolism, receptor overexpression, microenvironmental shifts—often precede detectable morphological changes; radioligands targeting these early alterations could reveal emerging malignancy or premalignant conditions before radiologically evident lesions appear [24,25]. In principle, repeated low-dose molecular PET could identify these early events.

In addition, a study demonstrated a transitioning from a conventional to LAFOV PET/CT system reduced the time patients were required to remain stationary during the scan and resulted in decreased patient concerns [26].

As defined the review of Slart et al. [10], the increased sensitivity of LAFOV-PET/CT systems can be leveraged to significantly reduce the administered radiotracer activity, thereby lowering patient radiation exposure and potentially enabling screening-oriented applications. The authors suggest in this context, how ultra-low-dose PET protocols may open new perspectives for targeted screening in high-risk populations, where early molecular alterations—such as preclinical amyloid deposition in neurodegenerative diseases or early oncologic and cardiovascular pathophysiological changes—precede clinical symptom onset by several years and where early detection could substantially improve therapeutic effectiveness and long-term outcomes [10].

If confirmed, such an approach could shift oncology toward earlier intervention, personalized risk stratification, and prevention, rather than awaiting overt disease. However, many critical conditions must be met before implementation: prospective validation showing that early detection improves clinically relevant outcomes (e.g., survival, quality of life), demonstration of acceptable false-positive rates, clear follow-up and management pathways, cost-effectiveness, equitable access, and ethical oversight.

In short: the hypothesis is plausible and technically supported, but requires rigorous clinical and epidemiological demonstration. PET-based early detection should be considered an aspirational vision—one now technically accessible but not yet clinically validated.

## 6. Challenges, Ethical Considerations, and a Translational Roadmap for Total-Body PET

Despite its considerable potential as a platform for systemic oncology and early detection, total-body PET raises important technical, clinical, organizational, and ethical challenges that must be addressed to ensure responsible translation into clinical practice. Quantitative robustness under low-dose and dynamic acquisition protocols remains a central issue: although total-body PET has demonstrated uniform sensitivity and spatial resolution across the extended field-of-view [5,6], quantitative metrics such as SUV, volumetrics, and kinetic parameters may still be affected by noise, scatter, randoms, dead-time losses, and reconstruction-related biases under ultralow-activity or rapid-scan conditions. Methodological analyses indicate that test–retest variability in ultralow-dose LAFOV PET/CT studies is driven predominantly by technical and biological factors rather than by pure statistical noise [23]; in particular, vendor-dependent list-mode resampling strategies based on sequential scan truncation may introduce subtle biases related to tracer uptake and clearance kinetics, especially in healthy organs. Additional sources of variability include patient-related metabolic factors, such as plasma glucose fluctuations, as well as heterogeneity in reconstruction and harmonization approaches—often reflecting incomplete adherence to EARL standards—which currently limits direct quantitative comparisons across published studies and underscores the need for harmonized acquisition and reconstruction protocols, standardized multicentre validation, and robust normative reference frameworks [23]. Beyond technical considerations, clinical interpretation of minimal or incidental uptake findings in asymptomatic individuals remains fraught with uncertainty, as focal tracer accumulation may reflect benign inflammatory processes, physiological variability, or non-malignant hyperplasia rather than incipient malignancy; in the absence of validated criteria for positivity and clinical significance, widespread PET-based surveillance carries a substantial risk of overdiagnosis and overtreatment, highlighting the need for carefully designed follow-up algorithms, consensus guidelines, and structured patient counselling. Accessibility and equity also represent critical concerns: while LAFOV PET/CT systems (>1 m) offer an order-of-magnitude gain in sensitivity over conventional PET/CT—enabling protocol optimization in terms of injected activity, acquisition time, and image quality—their high cost and limited availability may concentrate deployment in high-resource or academic centers, potentially exacerbating disparities in access. Nevertheless, real-world prospective observations of patient pathways before and after transition from conventional to LAFOV PET/CT systems demonstrate that implementation is feasible from both clinical and organizational perspectives, with significantly reduced uptake and scanning times, lower patient-reported anxiety and radiation-related concerns, and preserved levels of physical comfort and perceived quality of care, as assessed using standardized Likert-scale questionnaires [17].

In addition, ethical and regulatory frameworks must evolve in parallel with technological advances to ensure that the clinical deployment of PET-based screening or surveillance remains both scientifically sound and socially responsible. Beyond technical feasibility, such applications require robust and transparent informed consent procedures, clear communication of potential benefits and risks, long-term monitoring strategies, secure and accountable data governance, standardized approaches to the management of incidental findings, and shared consensus on follow-up pathways and intervention thresholds. In this context, the promise of early detection must be carefully balanced against the risks of overdiagnosis, psychological burden, and unnecessary downstream interventions. Addressing these multidimensional challenges demands a coordinated and forward-looking roadmap, integrating multicentre standardization and validation initiatives, prospective clinical trials in well-defined high-risk populations, and comprehensive evaluations of health outcomes and cost-effectiveness. Equally critical is the development of ethical, regulatory, and policy frameworks through sustained engagement of professional societies, regulatory authorities, public health stakeholders, and patient representatives, alongside the expansion of dedicated education and training programs for multidisciplinary teams. Finally, the establishment of interoperable, high-quality data infrastructures is essential to enable secure data aggregation, rigorous quality control, and long-term outcome monitoring—without which large-scale implementation of total-body PET risks generating unintended harm rather than meaningful population-level benefit.

Despite the extraordinary clinical and scientific advantages, the large-scale adoption of LAFOV PET technology must face significant economic barriers, involving not only the high initial investment for the equipment but also the potential substantial long-term costs associated with specialized maintenance and the complex infrastructure required.

While LAFOV-PET scanners represent a monumental leap in sensitivity and temporal resolution, they are not currently intended to render conventional PET/CT obsolete; rather, they serve as a specialized tool for ‘systemic’ evaluations—such as investigating complex multi-organ pathologies, performing ultralow-dose imaging for pediatric and longitudinal studies, and advancing pharmacokinetics for tracer development—while conventional PET/CT remains useful for routine clinical staging and high-throughput diagnostic workflows due to its established protocols and cost-effectiveness.

## 7. Discussion and Conclusions

The advent of total-body PET/CT represents one of the most significant technological improvements in the history of molecular imaging [27]. By overcoming the intrinsic sensitivity and coverage limitations of conventional PET systems, long-axial field-of-view scanners redefine not only how PET images are acquired, but also how PET can be conceptually positioned within modern oncology [28,29]. The combination of ultra-high sensitivity, whole-body coverage in a single bed position, and true dynamic total body imaging enables a level of systemic biological interrogation that was previously unattainable in routine clinical practice.

From a clinical perspective, total-body LAFOV-PET will consolidate and amplify the established strengths of PET/CT in cancer staging, restaging, and treatment response assessment. Improved lesion detectability, particularly for small-volume or low-uptake disease, may translate into more accurate staging and earlier identification of disease recurrence, of particular impact on PSMA- and SSTR-targeted imaging and on future research for new radiopharmaceuticals, such as radiolabeled monoclonal antibodies with long-lived radionuclides (e.g., 89Zr, 90Y), where radiation burden is a limiting factor [10]. At the same time, the ability to substantially reduce injected activity or acquisition time without compromising diagnostic quality offers tangible benefits in terms of patient comfort, scanner throughput, and cumulative radiation exposure—an especially relevant aspect in patients requiring repeated longitudinal imaging, in paediatric and pregnant patients [22].

Beyond incremental diagnostic gains, total-body PET introduces a qualitative shift toward truly quantitative and dynamic whole-body imaging. Simultaneous assessment of tracer kinetics across all organs opens new opportunities for pharmacokinetic modelling, individualized dosimetry, and therapy monitoring within theranostic frameworks [25]. In this context, total-body PET is not merely an imaging modality but an enabling platform that supports precision oncology by linking molecular target expression, treatment delivery, and biological response at the patient-specific level.

One of the most provocative implications emerging from this technological evolution is the potential extension of PET into domains traditionally considered beyond its scope, such as early cancer detection also in paediatric patients or surveillance in selected high-risk populations in view of low radiation exposure [30,31]. The feasibility of ultralow-dose whole-body PET challenges historical assumptions regarding radiation burden as a prohibitive limitation. Moreover, the biological rationale underpinning molecular imaging—namely that functional and molecular alterations precede anatomical changes—supports the hypothesis that PET could identify disease at an earlier, potentially more curable stage. Nevertheless, at present, this remains a hypothesis rather than an evidence-based indication. Robust prospective data demonstrating clinical benefit, acceptable specificity, and cost-effectiveness are still lacking, and the risk of overdiagnosis and downstream harm must be carefully weighed.

Importantly, the broader implementation of total-body PET also raises non-technical challenges. Quantitative harmonization across centers, standardization of acquisition and reconstruction protocols, and validation of biomarkers derived from ultralow-dose or dynamic imaging are prerequisites for widespread adoption. Furthermore, the high cost and limited availability of LAFOV systems raise legitimate concerns regarding equitable access and resource allocation, particularly if future applications extend beyond specialized oncologic indications. Ethical considerations—especially in the context of surveillance or incidental findings—must be proactively addressed through clear guidelines, informed consent processes, and multidisciplinary governance.

In conclusion, the convergence of new total-body PET technology and advanced radiopharmaceutical development marks a pivotal moment in molecular imaging and oncology. PET is evolving from a primarily diagnostic and staging tool into a comprehensive, systemic platform capable of supporting biological characterization, personalized therapy, and longitudinal disease monitoring. While the promise of earlier detection and preventive applications is a mirage, their potential realization will depend on rigorous scientific validation, thoughtful clinical integration, and responsible ethical oversight. If these conditions are met, total-body PET may play a transformative role in shaping a more proactive, precise, and biologically driven approach to cancer care.

## Data Availability

Not applicable.
